# Intraosseous Follicular Adenomatoid Odontogenic Tumour—A Case Report

**DOI:** 10.1155/2009/597483

**Published:** 2010-03-11

**Authors:** Farhan Durrani, Royana Singh

**Affiliations:** ^1^Department of Periodontics and Oral Surgery, Faculty of Dental Sciences, Institute of Medical Sciences, Banaras Hindu University, Varanasi 221005, India; ^2^Department of Anatomy, Institute of Medical Sciences, Banaras Hindu University, Varanasi 221005, India

## Abstract

The adenomatoid odontogenic tumour is a relatively uncommon lesion which mainly affects females in their second decade of life. It exhibits a predilection for the anterior region of the maxilla. The lesion is usually associated with the crown of an embedded tooth, most commonly the maxillary canine. In this paper, we present a case of adenomatoid odontogenic tumor affecting the left maxillary region in a 24-year-old female. The authors also discuss clinical, radiographic, histopathologic, and therapeutic features of the case.

## 1. Introduction

The adenomatoid odontogenic tumor (AOT) represents 3%–7% of all odontogenic tumors and was once considered as a variant of ameloblastoma. Microscopically, AOT exhibits tubular characteristic and duct-like structures that led to the term “adenoameloblastoma,” which previously used to designate this lesion [1]. In contrast to ameloblastoma, AOT is a circumscribed lesion with slow growth. A few extraosseous variants of AOT have been reported. In one of them the basal cells of oral epithelium were a potential source of origin [2]. In the latest edition of WHO classification of odontogenic tumors in 2005, AOT was classified into the first group of tumors (odontogenic epithelium without ectomesenchyme) instead of the second group (odontogenic epithelium with ectomesenchyme) [3]. Because of the absence of ectomesenchyme in immunohistochemical staining, dysplastic dentin, AOT is now considered the result of a metaplastic process rather than epithelial-ectomesenchyme interaction [4]. In this paper, a rare odontogenic tumor with histopathologic features resembling AOT along with hard tissue formation is reported. The Armed Forces Institute of Pathology (AFIP) in United States of America has described a similar neoplasm with recurrence potential and suggested the term “adenoid ameloblastoma with dentinoid” for these lesions [4]. A few other examples with different names have been reported in the literature.

## 2. Case Report

**Figure 1:**
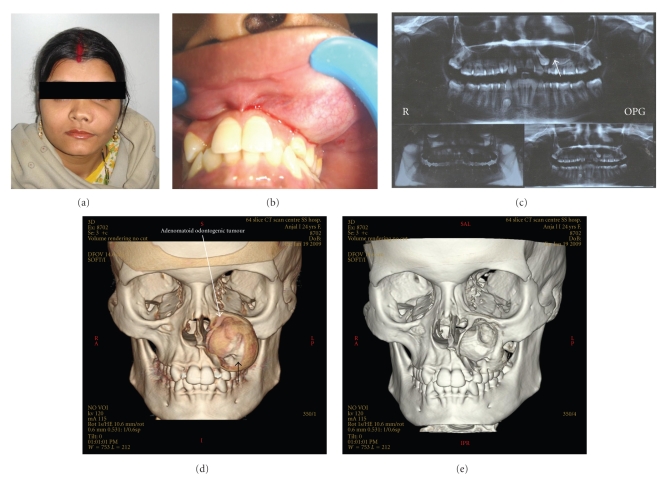
(a) External features of 22-year-old female exhibiting a swelling on the left cheek. (b) Swelling over the left anterior surface of the maxilla protruding on the upper left vestibule. (c) Oral Pantogram showing well defined radiolucent cyst with canine tooth (arrow). (d) and (f) Denta scan showing well circumscribed tumor with a tooth (arrow) covering the left maxilla.

**Figure 2:**
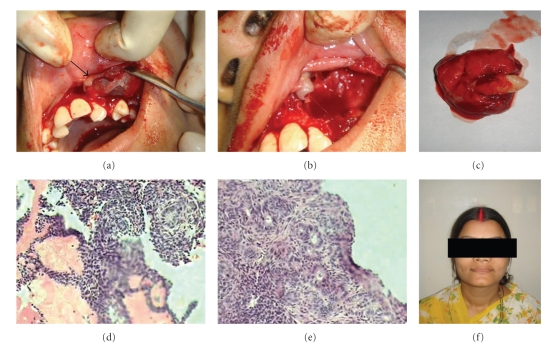
(a) and (b) showing the procedure undertaken for the removal of tumor, the tooth visible on removal of the mucosa (arrow). (c) The tumor with the canine tooth. (d) and (e) Microphotographs showing multiple cysts and stellate formation. (e) Photograph of the female patient completely asymptomatic after three months.

A 24-year-old Indian female was referred by her general practitioner for evaluation of a maxillary swelling to Department of Oral Medicine, Faculty of Dental sciences, Institute of Medical Sciences, Banaras Hindu University. The medical history was insignificant. The patient was asymptomatic and in good general health. Intraoral examination disclosed a nontender expansion of the left maxilla, covered by normal mucosa (Figures 1(a) and 1(b)). The patient had no nerve deficit or adenopathy in the face or neck. An orthopantomogram revealed the presence of a significant unilocular radiolucent area with well-defined sclerotic borders, involving an embedded upper left permanent canine (Figure 1(c)). A Denta scan (64 slice CT Scan) showed a well-defined tumour mass covering the complete left maxilla (Figures 1(d) and 1(e)). According to the clinical and surgical findings, the lesion was diagnosed as an odontogenic cyst. Enucleation of the lesion was performed, to completely extirpate the cystic lesion with extraction of upper left canine (Figures 2(a), 2(b), and 2(c)).

 The differential diagnosis was of dentigerous cyst, calcifying odontogenic cyst, calcifying epithelial tumor, odontogenic keratocyst, and unicystic ameloblastoma. Using local anesthesia, an excisional biopsy was performed. The surgical sample was fixed in formalin, embedded in paraffin, and stained with hematoxylin-eosin using the standard method. The tumor displayed a cystic pattern with characteristic features of a plexiform-type ameloblastoma, containing microcysts formation. Sheets and cords of epithelial cells were observed, which demonstrated a loose arrangement similar to stellate reticulum, intermixed with focal areas showing a whorled appearance. Reverse polarity of peripheral cells was prominent and tubular or duct-like structures lined by cuboidal cells were observed in some areas (Figures 2(d) and 2(e)). The patient was completely asymptomatic after 3 months (Figure 2(f)).

## 3. Discussion

The AOT is an uncommon cause of jaw swelling. There is a slight female over male incidence, almost 2 : 1, and appears most often in the second decade of life [5–7]. The sex and the age of the patient we described in this report are consistent with the literature. The lesions are typically asymptomatic, but may cause cortical expansion and displacement of the adjacent teeth [7]. The origin of the AOT is controversial [8]. Because of its predilection for tooth-bearing bone, it is thought to arise from odontogenic epithelium. The tumor has three clinico-pathologic variants, namely, intraosseous follicular, intraosseous extrafollicular, and peripheral. The follicular type (in 73% of all AOT cases) is associated with an unerupted tooth; whereas extrafollicular type (24%) has no relation with an impacted tooth [8]. Follicular and extrafollicular types are over two times more located in the maxilla than in the mandible and most of the tumors involve anterior aspect of the jaws [9]. In our case, the tumor was an intrafollicular intraosseous type and also found in the anterior region of the maxilla. Although larger lesions reported in the literature, the tumors are usually in the dimensions of 1.5 to 3 cm. Radiographically, they usually appear unilocular, may contain fine calcifications, and irregular root resorption is rare [10, 11]. This appearance must be differentiated from various types of disease, such as calcifying odontogenic tumor or cysts. The differential diagnosis can also be made with ameloblastoma, ameloblastic fibroma, and ameloblastic fibro odontoma [5]. The patient in the present report presented with no root resorption, but displacement of the adjacent teeth. It was also associated with an embedded tooth. Radiographically, it was easily differentiated from dentigerous cyst, which usually occurs as a pericoronal radiolucency. The histological findings for AOT are remarkably similar in the literature [6–8, 11]. The histological features of the tumor were described as a tumor of odontogenic epithelium with duct-like structures and with varying degree of inductive changes in the connective tissue. The tumor may be partly cystic and in some cases the solid lesion may be present only as masses in the wall of a large cyst. The tumor may contain pools of amyloid-like material and globular masses of calcified material. Our case was consistent with the common features reported in the literature [10, 11]. The tumor is well encapsulated and shows an identical benign behaviour. Therefore, conservative surgical enucleation produces excellent outcome without recurrence. Our patient has been under follow-up for 6 months.
